# Immunotherapeutic Value of Transcription Factor 19 (TCF19) Associated with Renal Clear Cell Carcinoma: A Comprehensive Analysis of 33 Human Cancer Cases

**DOI:** 10.1155/2022/1488165

**Published:** 2022-09-06

**Authors:** Xiaobao Cheng, Jian Hou, Xiangyang Wen, Runan Dong, Zhenquan Lu, Yi Jiang, Guoqing Wu, Yuan Yuan

**Affiliations:** Department of Urology, The University of Hongkong-Shenzhen Hospital, Shenzhen, China

## Abstract

**Background:**

We aimed to study the relationship between transcription factor 19 (TCF19) and cancer immunotherapy in the 33 types of human cancers.

**Methods:**

The Cancer Genome Atlas database was analyzed to obtain the gene expression data and clinical characteristics for the cases of 33 types of cancers. GSE67501, GSE78220, and IMvigor 210 were included in the immunotherapy cohorts. Relevant data were obtained by analyzing the gene expression database. The prognostic value of TCF19 was determined by analyzing various clinical parameters, such as survival duration, age, the stage of the tumor, and sex of the patients. The single-sample gene set enrichment analysis method was used to determine the activity of TCF19 and the method was also used to assess the differences between the TCF19 transcriptome and protein levels. The correlation between TCF19 and various immune processes and elements such as immunosuppressants, stimulants, and major histocompatibility complexes were analyzed to gain insights into the role of TCF19. The coherent paths associated with the process of TCF19 signal transduction and the influence of TCF19 on immunotherapy biomarkers have also been discussed herein. Finally, three independent immunotherapy methods were used to understand the relationship between TCF19 and immunotherapy response.

**Results:**

It was observed that TCF19 was not significantly influenced by the age (5/33), sex (3/33), or tumor stage (3/21) of cancer patients. But the results revealed that TCF19 exhibited a potential prognostic value and could predict the survival rate of the patients. In some cases of this study, the activity and expression of TCF19 were taken at the same level (7/33).

**Conclusion:**

TCF19 is strongly related to immune cell infiltration, immunomodulators, and immunotherapy markers. Our study demonstrated that high expression levels of TCF19 are strongly linked with the immune-related pathways. Nevertheless, it is noteworthy that TCF19 is not significantly associated with immunotherapy response.

## 1. Introduction

The renal tumor is one of the most common tumors in urology. Results from the statistical analysis conducted with the data associated with cancer revealed that renal tumors ranked second in terms of incidence of urinary system malignant tumors in China [1]. Clear cell renal cell carcinoma (ccRCC) is the major pathological type of renal cancer, which accounts for 70–80% of the cancers in urology. The annual percentage of increase in the rate of incidence is 3% in Europe and in the United States [2]. CcRCC is characterized as an aggressive tumor and approximately one-third of the patients suffering from ccRCC were diagnosed while tumor metastasis already occurred [3]. Cellular molecular-targeted therapy is the most effective method of treating metastatic ccRCC as patients suffering from kidney cancer do not respond to radiotherapy and chemotherapy. The European Urology Association (EUA) and the United States National Comprehensive Cancer Network (NCCN) recommended the molecular-targeted drugs as the first and second-line medicine for metastatic ccRCC [4, 5]. The prognostic factors of ccRCC include histological factors, tumor anatomical factors, molecular factors, and clinical factors. Among these, currently known molecular markers such as carbonic anhydrase 9, CRP [6, 7] , and cabozantinib [8] are not of high prognostic value and accuracy, and these have not been recommended for clinical application. At present, there are no universally accepted and reliable standard predictors for the diagnosis and prognosis of ccRCC at an early stage. The exploration of abnormally expressed genes in ccRCC tissues can potentially help identify new molecular biomarkers for the diagnosis and prognosis of ccRCC.

Transcription Factor 19 (TCF19) is a protein-coding gene that encodes a protein with a PHD-type zinc finger domain that is involved in transcriptional regulations [9]. At first, TCF19 was isolated from human, mouse, and hamster cells and it acts as a growth regulatory molecule [10]. TCF19 is associated with cell growth and regulation by affecting the G1S phase of the cell cycle. The genetic coding region of TCF19 is located on the short arm 6P21.3 of autochromosome 6, with a total length of 5.60 KB [11]. TCF19 is present in almost all human tissues, and its levels of expression are high in various tumor tissues [12–15]. Although current studies indicate that TCF19 may be associated with the progression of various tumors, few mechanisms have been reported for the role of TCF19 in carcinogenesis and immune regulation.

The processes of carcinogenesis and immune regulation are significantly affected by the physiological effects of TCF19 activation. Since TCF19 is chronically activated, it is highly expressed in various solid tumors [12–15] and chronic inflammatory tissues [16–18]. The presence of highly expressed TCF19 has been found not only in invasive tumor tissues but also in malignant tumor cell lines. This potentially indicates that TCF19 is correlated to the responses of inflammation and cell cycle progression [11, 16]. The genes associated with the TCF family regulate innate immunity and adaptive immunity [19, 20]. It has been previously reported that TCF1 helps achieve a balance between the CD8+ T cells by regulating the internal IL-10 signaling pathway which in turn influences immunotherapy [21]. Macrophages, a substantial component of the innate immune system, are related to the antitumor immune response in various cancers. It was stated that the M2 tumor-associated macrophages (TAMs) promote the processes of tumor progression, recurrence, and distal metastasis [22]. Macrophages are polarized by the stimulation of transcription factors in the tumor microenvironment by controlling their antitumor activity and by affecting their immunotherapy [23, 24]. Our previous study also confirmed that changes in macrophage polarization play substantial activities to regulate the inflammatory traumatic urethral stricture [25] and resistance to chemotherapy and endocrine therapy in advanced prostate cancer [26]. In general, TCF family genes significantly influence the immune system and the state of tumor tissue. Nevertheless, the immunotherapeutic value of TCF19 in the cases of human cancer has been rarely studied.

Herein, we described the expression profile of TCF19 in 33 different cancers and studied the potential regulatory roles of TCF19 for controlling the ccRCC immune microenvironment. Also, we studied the microsatellite instability (MSI) and tumor mutation burden (TMB) in ccRCC. Moreover, the association of the expression level of TCF19 with immune checkpoint blocking therapy was also investigated. In brief, this research provides data that help understand the immunotherapeutic role of TCF19 in ccRCC which may potentially help design various functional experiments.

## 2. Methods

**Figure 1:**
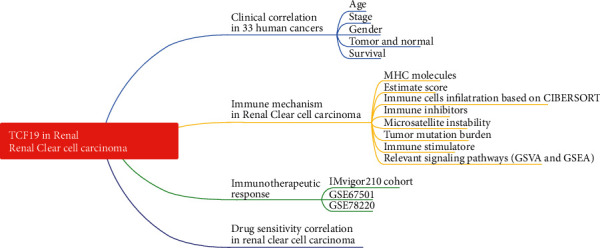
The flowchart of the study. Firstly, the expression of TCF19 is investigated within the different ages, stages, genders, and tissues, then the GSEA is utilized to explore the relevant immune signaling pathways based on the expression level of TCF19. Secondly, we apply the univariate Cox regression model and the Wilcoxon test between the nonresponder and responder groups of the immunotherapeutic response cohort to identify the survival association. Finally, we perform the drug sensitivity correlation with TCF19 expression in renal clear cell carcinoma.

(See Figure 1) shows the flowchart of this research.

### 2.1. Data Collection

The TCGA database (https://portal.gdc.cancer.gov/), a robust database, provides information on cancer genes. The database includes information on gene expression profiles, copy number variation (CNV), and single nucleotide polymorphism (SNP). We downloaded the mRNA expression and SNP data of 33 tumors for this study. Also, we downloaded the data from the GTEX database (https://commonfund.nih.gov/GTEx). Following the merging with the TCGA data and correction, we identified the differential expressions for various types of cancers. Moreover, we downloaded the corresponding tumor cell lines data from the CCLE database (https://portals.broadinstitute.org/ccle/), and we investigated the expression level of the gene in these tumor tissues. Furthermore, we investigated the significant correlation of this gene with the stages of tumor progression.

### 2.2. Association of TCF19 Expression with Clinical Characteristics of 33 Cancers

We downloaded the progression-free survival (PFS) and overall survival (OS) TCGA data of patients from the Xena database to evaluate the association of this gene with the prognosis of the patients. We utilized the Kaplan–Meier (K-M) method to analyze the survival curve (*P* < 0.05) for every cancer type. We employed “survival” and “SurvMiner” *R* packages for the survival analysis. Also, we used “survival” and “forest-plot”*R* packages for the Cox analysis to evaluate the interrelation of gene expression with the magnitude of survival of the patients.

### 2.3. TCF19 Enrichment Analysis

#### 2.3.1. Gene Set Variation Analysis (GSVA) Enrichment Analysis

GSVA, a package for the R program, was used to identify the enrichment of transcriptomic gene sets. GSVA identifies the changes from the level of the gene to the level of the pathway. This is achieved by using the specific gene sets of biological function. We utilized the Molecular Signatures Database (v7.0) for downloading the gene sets. GSVA algorithm identified the score of each gene set to determine the ability of changes in biological function within the different samples.

#### 2.3.2. Gene Set Enrichment Analysis (GSEA) Enrichment Analysis

In the GSEA analysis, we used predefined gene sets and sequencing gene sets (based on the differential expression level between the two types of samples). This method identifies whether the predefined gene sets were significantly enriched in the sequencing table. The “cluster profiler” and the “enrich-Plot” packages were used for the GSEA analysis and for exploring the imaginable mechanisms at the molecular level for the differential prognosis of different patients with different tumors. The differences in the signaling pathways associated with the high and low gene expression groups were studied, and the findings were compared.

#### 2.3.3. The Expression Level of TCF19 Is Correlated with Immune-Related Factors

RNA-seq data from patients with different subgroups of 33 cancers were analyzed by using the CIBERSORT algorithm to understand the content of infiltrating immune cells. This method also identifies the relation of gene expression with the content of immune cells. Moreover, we used the TISIDB website to identify the relation of gene expression with various immune factors, including chemokines, immune-stimulators, immune-suppressants, and MHC molecules.

#### 2.3.4. Correlation Analysis of TCF19 Expression and Tumor Mutation

The total number of mutations, including base substitutions, deletions, and insertions in tumor cells is called TMB. The frequency and number of variation/exon lengths were calculated for every sample tumor, and TMB was calculated by dividing the nonsynonymous mutation sites by the total length of the protein-coding region. The MSI of every TCGA sample was obtained from the data presented in previously published reports [27].

#### 2.3.5. Correlation Analysis of TCF19 Expression with Drug Sensitivity and Immunotherapy Response

The National Cancer Institute (NCI) listed the Cellminer database which contains the information on 60 cancer cells [1]. At present, the widely used database is the NCI-60 cell line with a broad range of cancer cell samples and it is used to investigate the anticancer drugs. In our study, we downloaded the NCI-60 drug sensitivity data and the RNA-seq gene expression data to evaluate the relations of gene expression with the sensitivity of antitumor drugs. The correlation analysis method was utilized to achieve the results. We considered a *P*value <0.05 for the statistical threshold.

We analyzed the immunotherapeutic response according to the previous method [2]. We used three independent immunotherapeutic cohorts in our present study. Usually, immunotherapeutic ways provided four outcomes, including complete response (CR), partial response (PR), progressive disease (PD), and stable disease (SD). We divided the patients into responders and nonresponders. Patients who had CR or PR signs were categorized as responders compared to the nonresponders, who had signs of SD or PD. We utilized the Wilcoxon rank-sum test to investigate the expression differences of TCF19 between the responder and the nonresponder groups.

#### 2.3.6. Statistical Analyses

R (version 4.0) was used for all statistical analyses. We calculated the hazard ratios (HRs) and 95% confidence intervals followed by applying the univariate survival analysis model. We applied the K-M survival analysis to investigate patient survival time. We divided the patients into the high gene expression level and the low gene expression level to arrive at the appropriate results. The statistical tests were bilateral, and we considered a *P*value <0.05 for the statistical threshold.

## 3. Results

### 3.1. Results of the Analysis of TCF19 Expression and Clinical Correlation in 33 Cancers

**Figure 2:**
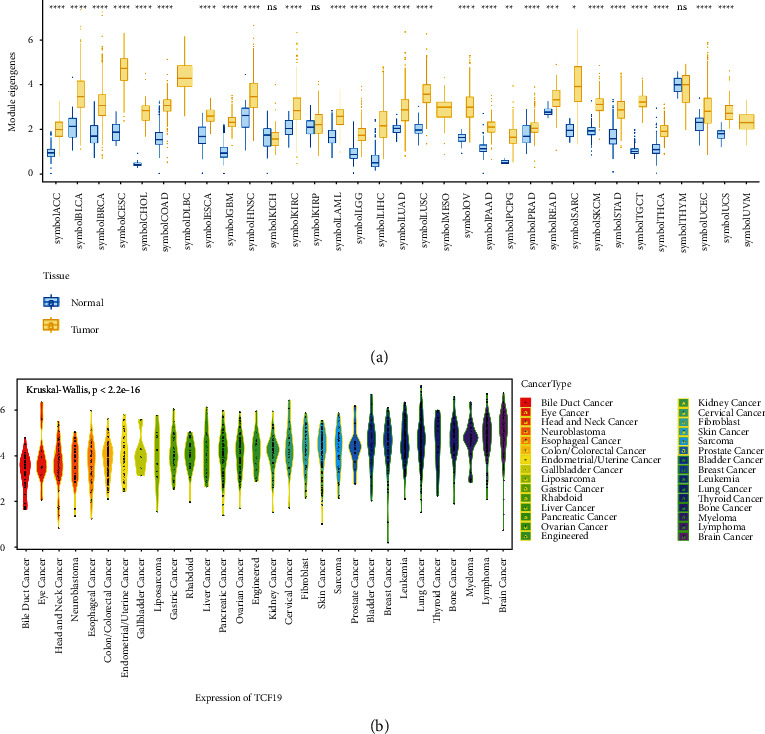
The expression of TCF19. (a) The TCF19 expression level in 33 human cancers using the TCGA combined with GTEx datasets and (b) the CCLE expression profile revealed that TCF19 is expressed in different tumor cell lines.

**Figure 3:**
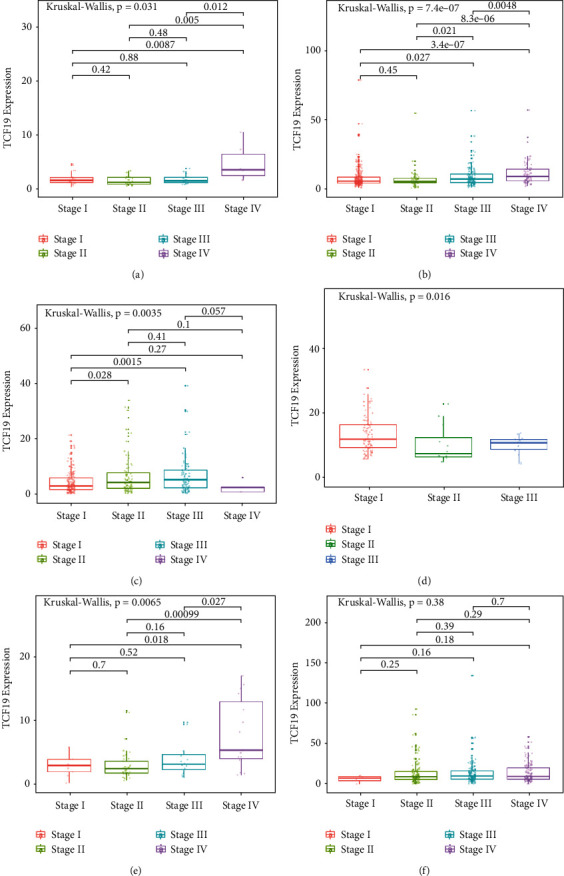
The correlation analysis of TCF19 with the stage of multiple tumors.

**Figure 4:**
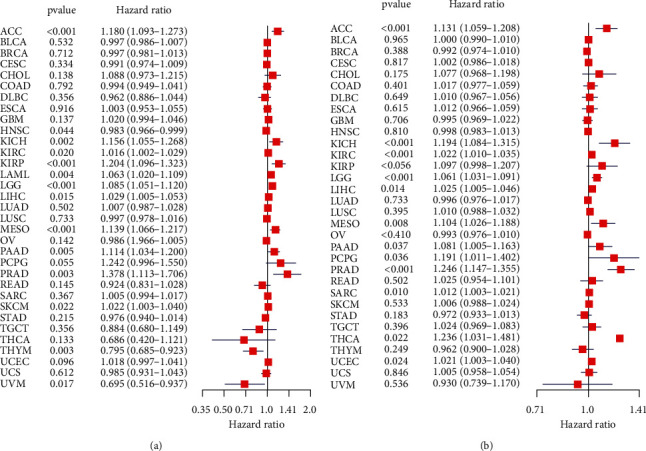
The association between TCF19 expression and prognosis of patients with multiple cancers. (a) The univariate regression model identifies the association of TCF19 expression with the overall survival (OS) rate in multiple cancer patients and (b) the univariate regression model identifies the association of TCF19 expression with the progression-free interval (PFI) of patients with multiple cancers.

We analyzed the expression level of TCF19 in 33 types of human cancers using the data presented in the TCGA and GTEX datasets.  Table 1 presented the full names of the 33 cancer types utilized in this comprehensive study. The high levels of expression of the gene were observed in 27 types of carcinomas, including ACC, BLCA, BRCA, CHOL, CESC, COAD, ESCA, GBM, HNSC, KIRC, LAML, LGG, LIHC, LUAD, LUSC, OV, PCPG, PAAD, PRAD, READ, SARC, SKCM, STAD, TCGT, THCA, UCEC, and UCS (Figure 2(a)). TCF19 expression levels in most normal tissues were lower than that in cancer cells. In the CCLE expression profile of various cell lines, the expression level of TCF19 is illustrated in figure 2(b). Moreover, we found that TCF19 expression was related to the stages of various tumors, such as ACC, BRCA, TGCT, KICH, KIRC, and LIHC (Figure 3). This work studied the correlation between the expression levels of TCF19 and survival prognosis in patients suffering from cancer. We found that the expression level of TCF19 was closely associated with the OS of patients in 14 different types of cancers (such as KIRC, ACC, KICH, KIRP, LAML, THYM, LGG, HNSC, LIHC, MESO, PRAD, SKCM, UVM, and PAAD; Figure 4(a)). In addition, the results from the KM-curve survival analysis suggested that the highly expressed TCF19 was correlated with poor OS in 13 types of malignant cancers, including ACC, BRCA, KICH, LIHC, GBM, SKCM, KIRC, KIRP, LGG, LUAD, PAAD, PCPG, and MESO (Supplementary Figure 1). The expression level of TCF19 was closely linked with PFI in 12 cancer types, including PAAD, ACC, MESO, KICH, LIHC, PCPG, PRAD, LGG, SARC, THCA, KIRC, UCEC, and other tumors (Figure 4(b)). The K-M curve analysis for survival prognosis suggested that a highly expressed group of TCF19 was associated with a shorter PFI in 10 kinds of malignant cancers (such as UCEC, ACC, KICH, PAAD, KIRC, LGG, LIHC, PCPG, PRAD, and THCA; Supplementary Figure 2).

**Figure 5:**
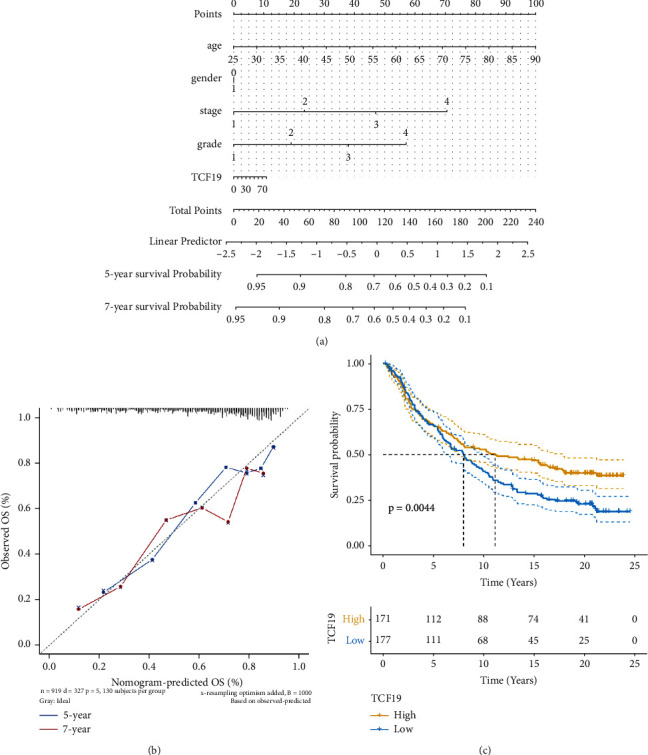
The TCF19 expression level is associated with the risk and prognosis of patients. (a) It shows the gene correlation column line graph model for TCF1, (b) it shows the correction curves plotted for two periods of five and seven years, and (c) it shows the Kaplan–Meier survival analysis plots of TCF19 expression versus patients treated with PD-L1.

A nomogram prediction model was constructed using the TCF19 expression level and the clinical features. The results obtained from regression analysis were displayed in the form of alignment charts. Variables such as gender, age, tumor stage, and grade were analyzed, and the results were presented. The gene correlation column diagram model of TCF19 of the constructed TCGA-KIRC sample is shown in Figure 5(a). Correction curves corresponding to the two periods were generated in the fifth and seventh years. The model effect was quite consistent (Figure 5(b)).

### 3.2. The TCF19 Expression Is Potentially Associated with Immune-Associated Factors

**Figure 6:**
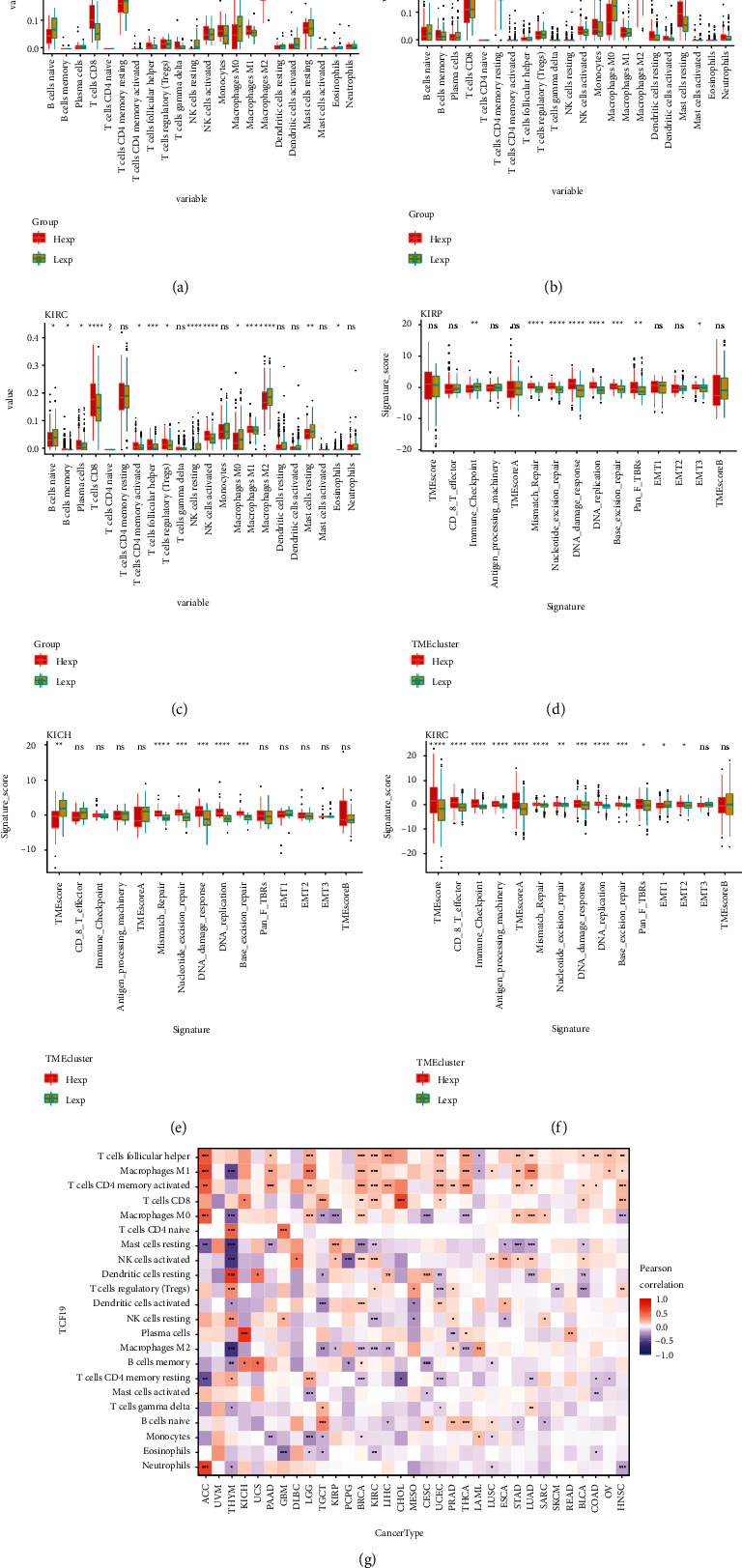
The TCF19 is correlated with the immune infiltration in pan-cancer. (a–f) The expression level of TCF19 is significantly correlated with the infiltration of immune cells in multiple cancers and (g) it indicates the correlation analysis of TCF19 expression with multiple tumors.

Tumor-associated fibroblasts, extracellular matrix, immune cells, various growth factors, inflammatory factors (characterized by special physicochemical characteristics), cancer cells, etc., are present in the tumor microenvironment. The microenvironment significantly affects the diagnosis of tumors, survival outcome, and degree of the response generated toward clinical treatment. Our findings indicated that the TCF19 expression level was substantially correlated with the infiltration of immune factors. TCF19 expression level was significantly related to the CD4 memory-activated cells in 14 kinds of cancers. In 15 kinds of cancers, the TCF19 expression level was significantly related to the follicular helper cells, and in the other 14 kinds of cancers the TCF19 expression level were correlated significantly with the macrophages M1 cell (Figure 6). Further analysis of the tumor microenvironment in kidney carcinoma (KIRC) revealed that TCF19 expression level was significantly related to the various gene set scores including the CD_8_T effector, TME score A, TME score, DNA damage response, base excision repair, immune checkpoint, antigen processing machinery, mismatch repair, nucleotide excision repair, DNA replication, Pan F TBRs, EMT1, and EMT2 in kidney carcinoma ().

### 3.3. GSVA/GSEA Correlation Analysis of TCF19

**Figure 8:**
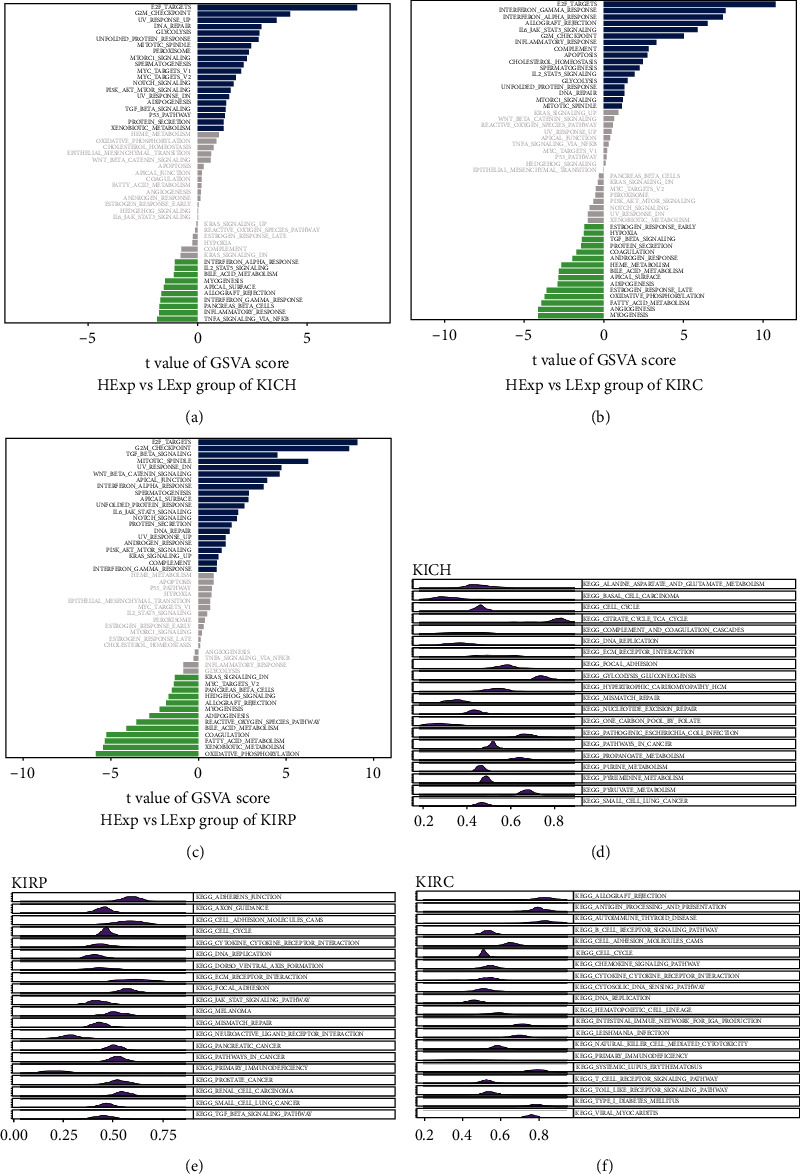
The results of GSVA analysis of TCF19. (a–c) It shows the GSVA analysis of TCF19 in KIRC, KIRP, and KICH, and (d–f)represents the GSEA analysis of TCF19 in KIRC, KIRP, and KICH.

The GSVA scores were determined for all tumors to elucidate the molecular mechanism associated with the TCF19 gene associated with pan-cancer. We divided the tumor samples into two groups based on the higher expression level and the lower expression levels. The median value of the gene expression level in each tumor was utilized for comparison. It was observed that in the case of kidney carcinoma, highly expressed TCF19 genes were primarily associated with some specific pathways such as interferon alpha response, E2F targets, allograft rejection, IL-6-JAK-STAT3 signaling, interferon gamma response, and G2M checkpoint (Figure 8(a)–8(c)). Results from the GSEA analyses of TCF19 and kidney carcinoma are presented in Figures 8(d)–8(f).

### 3.4. Correlation Analysis of TCF19 Expression with Tumor Mutations and Gene Regulation

The study further constructed the WGCNA net based on the KIRC expression profile for exploring the coexpression network linked with TCF19 in pan-cancer. The clustering chart of patients is shown in Supplementary Figure 3. We utilized the “soft power Estimate” function in the WGCNA package to identify the soft threshold *β* value and the value of *β* is set to 12. We detected 17 gene modules using the Tom matrix. These are black (298), blue (519), brown (446), cyan (357), green (354), green yellow (489), grey (3788), grey60 (82), light cyan (129), light green (74), light yellow (57), night blue (155), pink (449), purple (230), red (308), turquoise (1822), and yellow (443) (Supplementary Figure 3). The modules and traits were further analyzed, and it was found that the maximum correlation was observed for the ME green yellow module (COR = 0.35, *P* = (5E-19)) (Supplementary Figure 3). The coexpression analysis method was further used to explore the relationship between the level of TCF19 expression and 33 tumor immune-related genes. The analyzed genes included genes associated with MHC, immune activator, chemokine receptor proteins, immunosuppressor, and chemokine. It was observed that TCF19 was significantly associated with most of the immune-related genes (Supplementary Figure 4). Moreover, TCF19 was significantly associated with the crucial tumor-related marker genes that controlled the various biological processes, including the TGF beta signaling pathway, TNFA signaling, hypoxia, coking death, repair of DNA, autophagy, and ferroptosis (Supplementary Figure 5).

**Figure 9:**
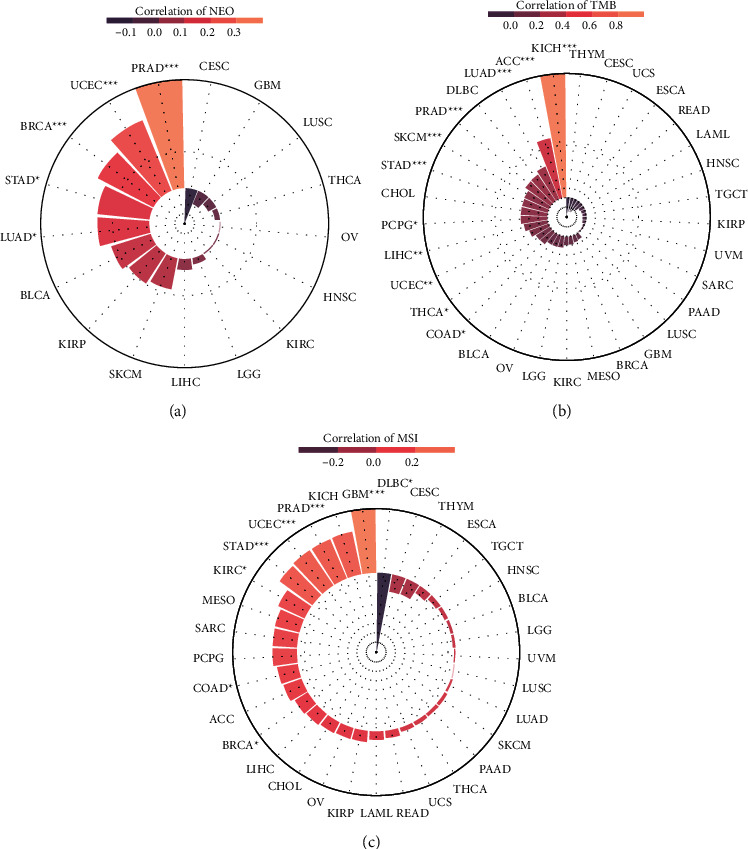
The relationship of TMB and MSI with the TCF19 expression in cancers. (a) Shows the relationship between TCF19 expression and TMB, (b) indicates the relations of TCF19 expression with MSI, and (c) represents the correlations of TCF19 expression with Neoantigen.

The immunotherapy response was crucially associated with some biomarkers, including TMB and MSI. We investigated the relation of TCF19 expression level with TMB in this study. We revealed that the TCF19 expression level was significantly correlated with TMB in all tumors, including P ACC, CPG, UCEC, SKCM, COAD, PRAD, STAD, KICH, LIHC, LUAD, and THCA (Figure 9(a)). A significant difference was observed for MSI in various cancers, including UCEC, KIRC, GBM, COAD, BRCA, STAD, PRAD, and DLBC (Figure 9(b)).

### 3.5. Correlation Analysis of TCF19 Expression with Drug Sensitivity and Immunotherapeutic Response

The effect of surgery and chemotherapy on the conditions of early-stage tumors had been widely explored. We investigated the cell miner database to identify the association of TCF19 expression level with IC50 values of antitumor drugs. We revealed that the higher expression level of TCF19 was correlated with the tolerance level of multiple antitumor drugs (Supplementary Figure 6). It was observed that TCF19 correlated positively with fludarabine, 6-mercaptopurine, dexamethasone decadron, nelarabine, and fenretinide. The gene negatively correlated with AFP464, trametinib, aminoflavone, cobimetinib (isomer 1), palbociclib, and lificguat.

The dataset corresponding to IMvigor 210 tumor immunotherapy was downloaded and 348 patients subjected to the conditions of PD-L1 therapy (and presenting complete survival information) were enrolled. The K-M survival analysis was used for the studies, and the results revealed that high TCF19 expression levels reflected the poor prognosis of patients (figure 5(c)).

## 4. Discussion

In China, kidney carcinoma is the second-highest malignant tumor in urology [1]. Approximately 1/3^rd^ of the patients developed metastatic carcinoma before diagnosis [5]. Advanced renal clear cell carcinoma showed resistance to the treatment strategies including radiotherapy and chemotherapy. Hence, the cellular and molecular-targeted treatment method is widely used to treat ccRCC. Multiple guidelines recommend molecular-targeted therapy as the first and second choice of treatment for metastatic ccRCC [6, 7]. Therefore, it is important to explore new therapeutic targets for advanced ccRCC.

At the beginning of the research, we identified the expression differences of TCF19 in tumor tissues relative to the normal samples. The results helped identify the potential immunotherapeutic value of TCF19. TCF19 is a gene that is associated with cell growth regulation which primarily regulates the cell cycle and the process of apoptosis. TCF19 was first isolated from mouse, human, and hamster cells. The previous report indicated that the TCF19 expression level was higher in various cancerous tissues, including the liver, colon, rectum, head and neck, lung, and gastrointestinal tract [12–15]. In this work, TCF19 was highly expressed in ACC, BLCA, KIRC, PRAD, TCGT, and other urinary system tumors which were under previous findings. In addition, the results from the K-M survival investigation suggested that a higher expression level of TCF19 is significantly associated with a shorter prognosis of various tumors in both OS and PFI. These studies might suggest that TCF19 is crucially linked with a shorter prognosis of multiple tumors.

Since TCF19 significantly affects the tumor immune microenvironment, more studies need to be conducted on the immune cells, tumor microenvironment, immunomodulators, and immunotherapy responses to gain in-depth knowledge. This study aimed to gain insights into the underlying mechanisms associated with the TCF19 gene that was associated with immune-related factors. 33 types of human cancers were studied to obtain relevant information. This work also aimed to explore the immune-related mechanisms associated with urinary tumors. The expression of TCF19 and clinical characteristics was analyzed, and the results obtained from COX regression analysis revealed that TCF19 was a prognostic factor of ccRCC. Correction curves were generated for the ccRCC patients in the fifth and seventh years and the consistent model effects were observed. Daniela Ruggiero reported the increased level of expression of the TCF19 gene in two major histological subtypes (squamous cell carcinoma (SCC) and lung adenocarcinoma) and revealed that TCF19 promoted the progression of the cell cycle in NSCLC cells. This validated the fact that TCF19 was a therapeutic target [28]. Du WB reported that TCF19 was significantly upregulated in colorectal cancer and TCF19 was closely related to the progression of malignancy, distant metastasis, and poor prognosis of colorectal cancer. So, he speculated that TCF19 could aggravate the malignant progression of CRC [29]. Ji, Xu, and Miao further reported that TCF19 was highly expressed in cancer cells associated with head and neck SCC, liver cancer, and gastric cancer. They reported that TCF19 could be potentially correlated with tumor prognosis by conducting gene assays, K–M survival analysis, and western-blot tests [12, 13, 15]. It is worth noting that the results of our research reflected the association of the gene with a substantial prognosis of these tumors and confirmed the reliability of the analytical results obtained. Moreover, the correlation between TCF19 and the prognosis of ccRCC was also reported. But now the mechanism involving TCF19 in the occurrence of ccRCC has not been clearly described. We may infer that the modulation of the TCF19 activity associated with ccRCC could potentially help obtain results that can help improve the therapeutic techniques.

Conventional surgical treatment and radiotherapy and chemotherapy cannot be effective to treat patients suffering from late-stage ccRCC. Maybe more research should be conducted on the gene targets and immune checkpoint inhibitors associated with pan-cancer as the results can potentially help predict the prognosis of antitumor immunotherapy. This research studied the relation of TCF19 with the process of immune cell infiltration for further investigating the crucial immunotherapeutic potential of TCF19. The results revealed that the expression level of TCF19 significantly correlated with the infiltration of the immune cells, including CD4 memory T cells, T follicular helper cells, and M1 macrophages. Analysis of the relationship between tumor microenvironment and KIRC revealed that KIRC was significantly correlated with some scores such as TMEscoreA, TMEscore, mismatch repair, CD8^+^ T effector, immune checkpoint, antigen processing machinery, nucleotide excision repair, and DNA damage. The scores of the responses, Pan F TBRs, DNA replication, base excision repair, EMT1, and EMT2 significantly correlated with KIRC. And this study further investigated the relations of TCF19 with the immune-related genes, including genes associated with MHC, immune activator, immuno-suppressive markers, chemokine, and their receptor protein. Interestingly, we found that immune-associated factors were significantly correlated with the expression level of the TCF19 gene. Our previous study reported that several immune-prognostic genes influenced the process of immunotherapy associated with urinary bladder cancer [30]. Besides, it has been reported that the regulation of macrophage polarization attenuated the inflammatory traumatic urethral stricture in New Zealand rabbits [25]. Another study recently reported that M2-tumor-associated macrophages (TAMs) were able to promote the process of bone metastasis and were able to influence the chemotherapy and drug resistance ability of the cells of prostate cancer. The regulation of the process of macrophage polarization can influence the effect of immunotherapy in patients suffering from prostate cancer [26]. Sen, Yang GH, and Mondal reported that TCF19, a novel pancreatic islet regulator, regulated the processes of energy metabolism and stress adaptation associated with the tumor cells by regulating gluconeogenesis. It was associated with the inflammatory responses in the beta cells of the pancreas and the DNA damage response network. The occurrence and progression of pan-cancer were also affected [16–18]. It has been reported recently that TCF19 influences the effect of immunotherapy in lung cancer through nanotechnology by regulating the polarity of the tumor-associated macrophages [31]. Those results revealed that TCF19 might influence the process of immunotherapy by regulating the immune-related genes and the inflammatory cells such as macrophages associated with tumor cell immunotherapy.

Furthermore, we observed that two immunotherapy biomarkers (TMB and MSI) were associated with TCF19 in various tumors. In general, as the number of somatic mutations in a tumor increase, the ability to generate neoantigens increases. It was also observed that the tumor neoantigen load could be efficiently determined by analyzing the TMB [32]. MSI is a robust mutant factor phenotype, the generation of which can be attributed to the presence of defects in mismatch repairing of DNA. MSI is a crucial predictor for immunotherapy responses [33]. This study showed that TMB and MSI were significantly associated with the TCF19 expression level in various tumors. However, the TCF19 expression level was not significantly associated with immunotherapy responses. Despite all 3 cohorts responded to antiPD1 therapy. We hypothesized that TCF19 might influence the extent of the response generated toward immunotherapy by targeting the various immune checkpoints. Also, our study only analyzed 3 relevant cohorts, which makes it difficult to elucidate the actual immunotherapy response of TCF19. More relevant immunotherapy cohort studies should be conducted in the future.

And finally, we followed the gene enrichment analysis to arrive at the result which revealed that the highly expressed TCF19 gene was primarily associated with specific pathways such as E2F, IL6, and G2M. The E2F and IL6 families are classical tumor signaling pathways. It has been reported that they exhibit unique and overlapping properties during the processes of transcription, proliferation, and apoptosis of tumor cells [34, 35]. The results might indicate that TCF19 potentially affects the extent of proliferation, infiltration, and metastasis realized by regulating multiple classical signaling pathways.Also, this specific mechanism associated with the processes needs to be explored further. The Cellminer database was analyzed to determine the relationship between TCF19 and IC_50_ to explore the correlation between TCF19 and antitumor drug sensitivity. The results revealed that the high level of expression of TCF19 reflected the tolerance level toward multiple antitumor drugs. The factors and mechanisms affecting the sensitivity of antitumor drugs are complex and diverse but results from the analysis of the K-M survival plot revealed that the higher expression group of TCF19 was significantly linked with a shorter prognosis for cancer patients. It was also observed that TCF19 negatively correlated with the effect of immunotherapy. The results indicated that TCF19 can be used as a potential indicator of the extent of the response generated toward renal cancer immunotherapy. Cancer immunotherapy based on TCF19 can also be explored and the results can potentially open a new avenue for the development of tumor immunotherapy strategies. For example, Han [36] predicted the clinical outcome when patients suffering from lung adenocarcinoma were subjected to conditions of radiotherapy and immunotherapy by analyzing the genetic characteristics of the B cells. Dai [37] constructed an immune-related gene prognostic index (IRGPI) based on 11 immune-related genes, which can accurately forecast the immune cell infiltrations in the tumor microenvironment of hepatocellular carcinoma and the response generated toward immunotherapy. Feng Xu [38] studied lung adenocarcinoma cases and reported that immune-related genes were independently predicting the poor survival rate of patients.

As per we know, there is a minor number of relevant researches currently available to explain the functions of TCF19 in ccRCC. This study provided valuable information on how the TCF19 gene participated in cancer immunotherapy. The results also revealed the relationship between TCF19 and various immune indicators (such as the infiltration process of immune cells, immune-modulatory factors, and the biomarkers of the immune system). The obtained data can potentially help understand the underlying mechanisms associated with TCF19 and the immune system. Although the correlation between tumor immune microenvironment and TCF19 cannot be applied to all kinds of tumors, our work revealed the immune effects of TCF19 on the microenvironment of specific cancer cells which may potentially help improve the processes of TCRCC targeting therapy. However, preliminary results have been reported using various bioinformatics methods. Therefore, further research should be conducted to understand how TCF19 influences cancer immunotherapy. In our next step, we need to extend the existing analysis database and mutually authenticate with the existing database. Authentication should be realized at the molecular, cytological, and animal levels by conducting experiments to investigate the relationship between the prognosis of the patients and the properties of the clinical tumor tissue samples. We believe that the results can potentially help for improving the efficiency of diagnosis, treatment methods, and survival prognosis of cancer patients.

## 5. Conclusion

This is one of the few studies that focus on the immunotherapeutic value of TCF19 associated with ccRCC. We believe that the results reported herein can potentially help design functional experiments that can help develop the field of clinical treatment.

## Figures and Tables

**Figure 7:**
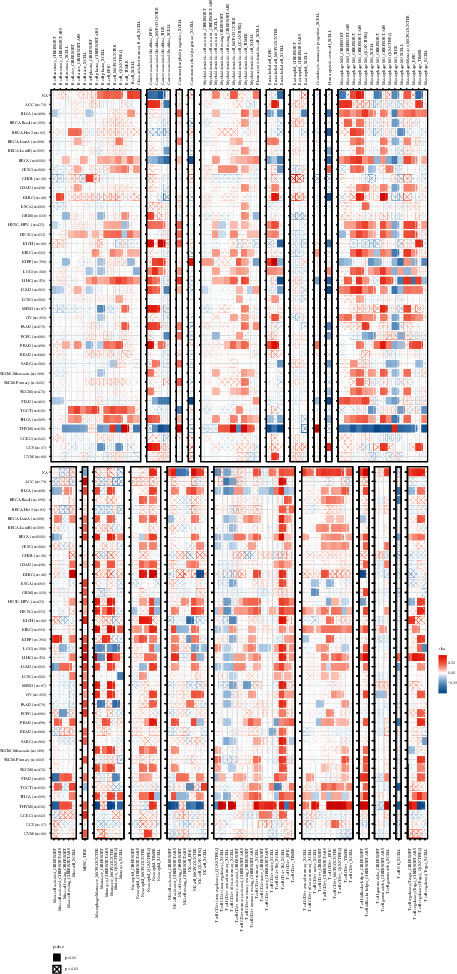
The analysis of TCF19 expression and the tumor microenvironment in the ccRCC.

**Table 1:** 33 types of human cancer studied in this research.

Abbreviation	Full name
ACC	Adrenocortical carcinoma
BLCA	Bladder urothelial carcinoma
BRCA	Breast invasive carcinoma
CESC	Cervical squamous cell carcinoma and endocervical adenocarcinoma
CHOL	Cholangiocarcinoma
COAD	Colon adenocarcinoma
DLBC	Lymphoid neoplasm diffuse large B-cell lymphoma
ESCA	Esophageal carcinoma
GBM	Glioblastoma multiforme
HNSC	Head and neck squamous cell carcinoma
KICH	Kidney chromophobe
KIPAN	Pan-kidney cohort (KICH + KIRC + KIRP)
KIRC	Kidney renal clear cell carcinoma
KIRP	Kidney renal papillary cell carcinoma
LAML	Acute myeloid leukemia
LGG	Brain lower grade glioma
LIHC	Liver hepatocellular carcinoma
LUAD	Lung adenocarcinoma
LUSC	Lung squamous cell carcinoma
MESO	Mesothelioma
OV	Ovarian serous cystadenocarcinoma
PAAD	Pancreatic adenocarcinoma
PCPG	Pheochromocytoma and paraganglioma
PRAD	Prostate adenocarcinoma
READ	Rectum adenocarcinoma
SARC	Sarcoma
STAD	Stomach adenocarcinoma
SKCM	Skin cutaneous melanoma
STES	Stomach and esophageal carcinoma
TGCT	Testicular germ cell tumors
THCA	Thyroid carcinoma
THYM	Thymoma
UCEC	Uterine corpus endometrial carcinoma
UCS	Uterine carcinosarcoma
UVM	Uveal melanoma

## Data Availability

The original data are provided by the corresponding author upon request without any hesitation.

## Abbreviations

BCa: Bladder cancer
TCGA: The cancer genome atlas
diff-LncRNAs: Differentially expressed LncRNAs
DEGs: Differentially expressed genes
GO: Gene ontology
KEGG: Kyoto encyclopedia of genes and genomes
CC: Cellular component
MF: Molecular function
BP: Biological process
FC: Fold-change
GSEA: Gene set enrichment analysis
DAVID: The database for annotation Visualization and Integrated Discovery
OS: Overall survival
ssGSEA: Single-sample gene set enrichment analysis.

## Supplementary Materials

Supplementary Materials Supplementary figure 1: the relationship between TCF19 expression and prognosis (OS) of cancer patients. Supplementary Figure 2: the relationship between TCF19 expression and prognosis of cancer patients (PFI). Supplementary Figure 3: the WGCNA analysis of TCF19 in pan-cancer. Supplementary figure 4(a–f): the relationship between TCF19 expression and 33 tumor immune-related genes (genes analyzed include MHC, immune activators, immune suppressors, chemokines, and chemokine receptor proteins). Supplementary Figures 5(a–g): the association between TCF19 and common tumor-associated regulatory genes (such as TGF beta signaling, TNFA signaling, hypoxia, scorch death, DNA repair, autophagy genes, and iron death-related genes). Supplementary figure 6: the analysis of the relationship between TCF19 and the sensitivity of common antitumor drugs.
