# Supplementary material for: Immunotherapeutic Value of Transcription Factor 19 (TCF19) Associated with Renal Clear Cell Carcinoma: A Comprehensive Analysis of 33 Human Cancer Cases
Source: J Oncol. 2022 Sep 6;2022:1488165. doi: 10.1155/2022/1488165 (PMC9470357; doi:10.1155/2022/1488165)
Supplement: Supplementary Materials — Supplementary figure 1: the relationship between TCF19 expression and prognosis (OS) of cancer patients. Supplementary Figure 2: the relationship between TCF19 expression and prognosis of cancer patients (PFI). Supplementary Figure 3: the WGCNA analysis of TCF19 in pan-cancer. Supplementary figure 4(a–f): the relationship between TCF19 expression and 33 tumor immune-related genes (genes analyzed include MHC, immune activators, immune suppressors, chemokines, and chemokine receptor proteins). Supplementary Figures 5(a–g): the association between TCF19 and common tumor-associated regulatory genes (such as TGF beta signaling, TNFA signaling, hypoxia, scorch death, DNA repair, autophagy genes, and iron death-related genes). Supplementary figure 6: the analysis of the relationship between TCF19 and the sensitivity of common antitumor drugs. [file 1488165.f1.docx]

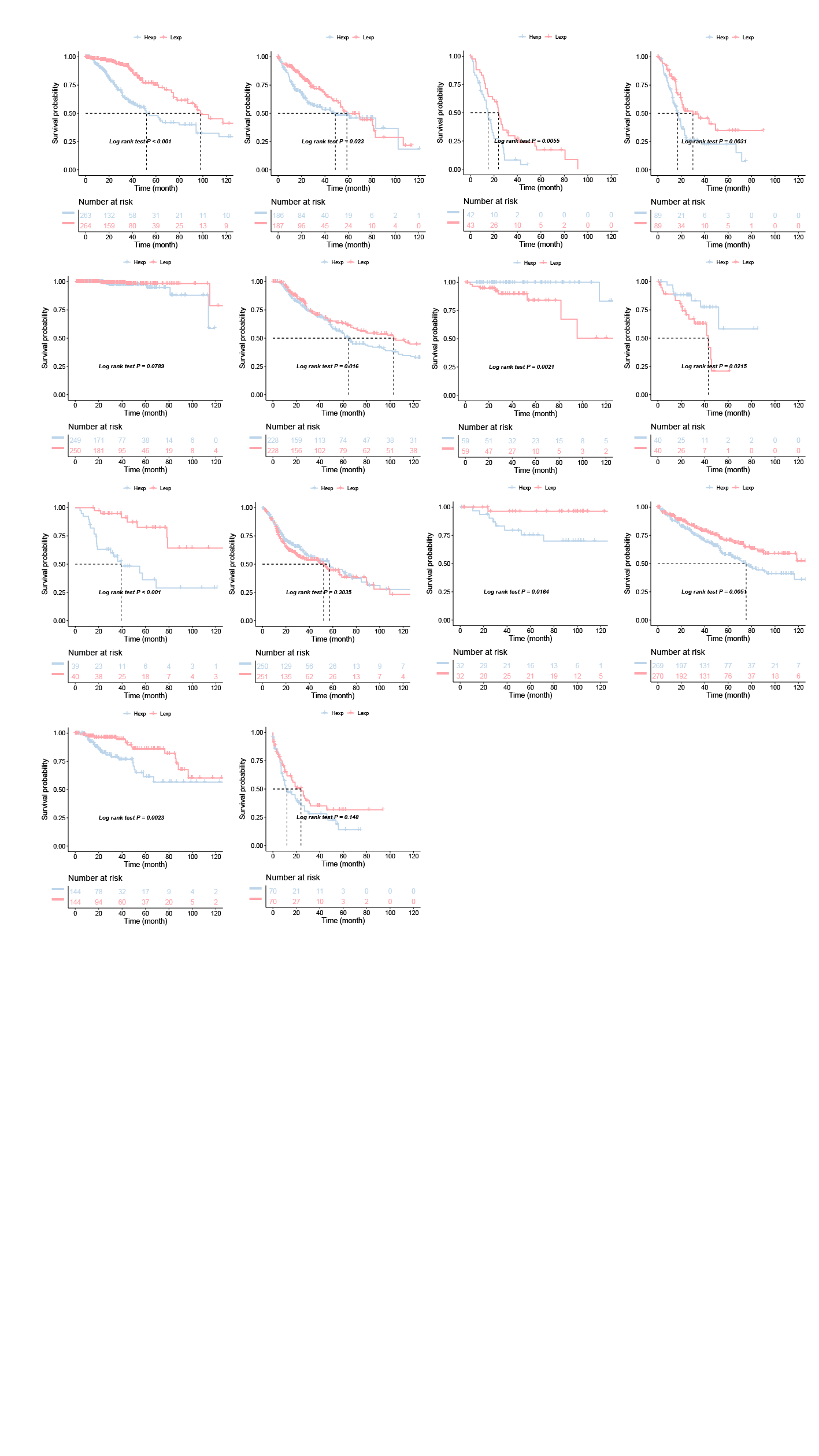


**SUPPLEMENTARY FIGURE 1** The relationship between TCF19 expression and prognosis (OS) of cancer patients.


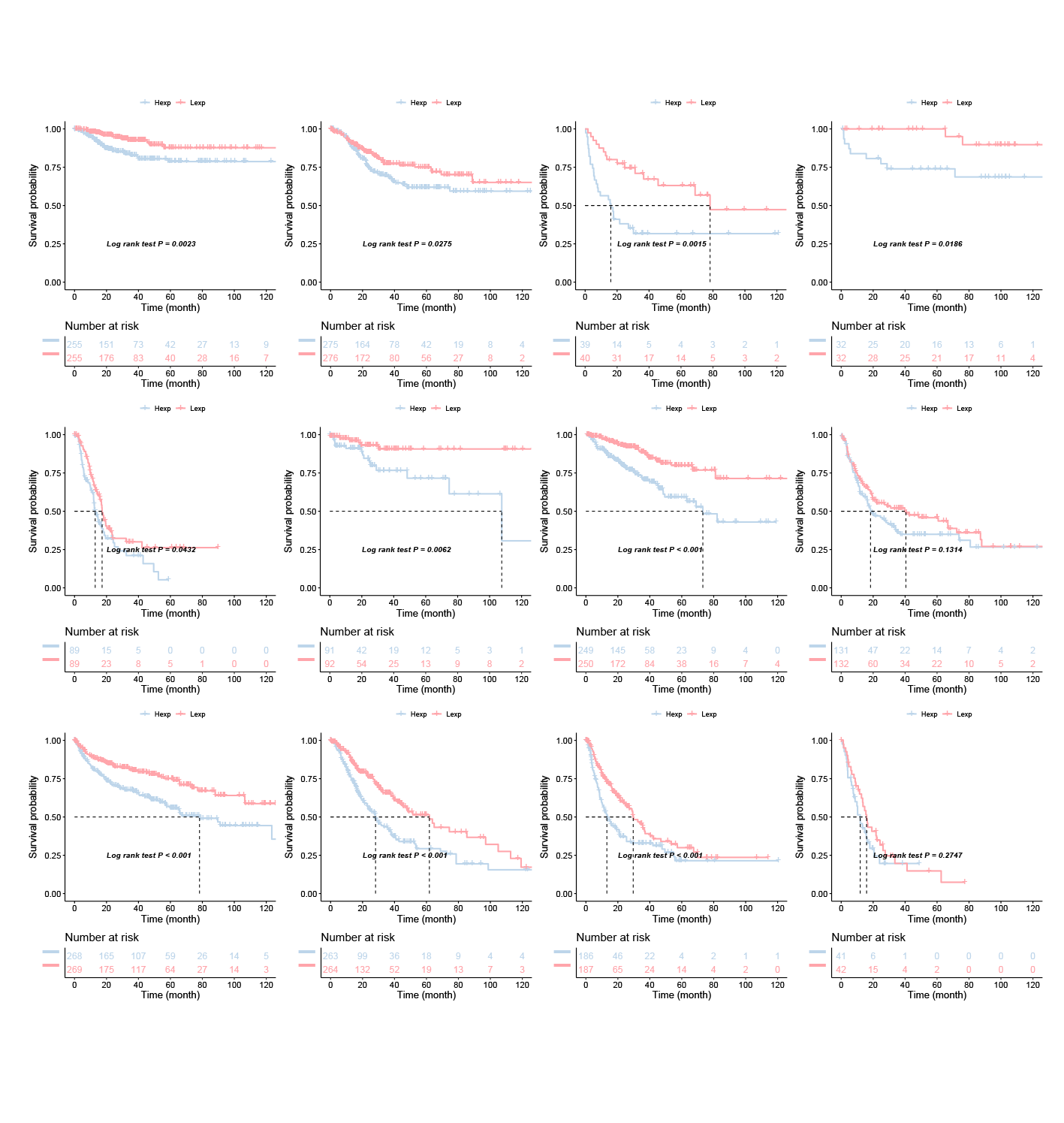


**SUPPLEMENTARY FIGURE 2** The relationship between TCF19 expression and prognosis of cancer patients (PFI).


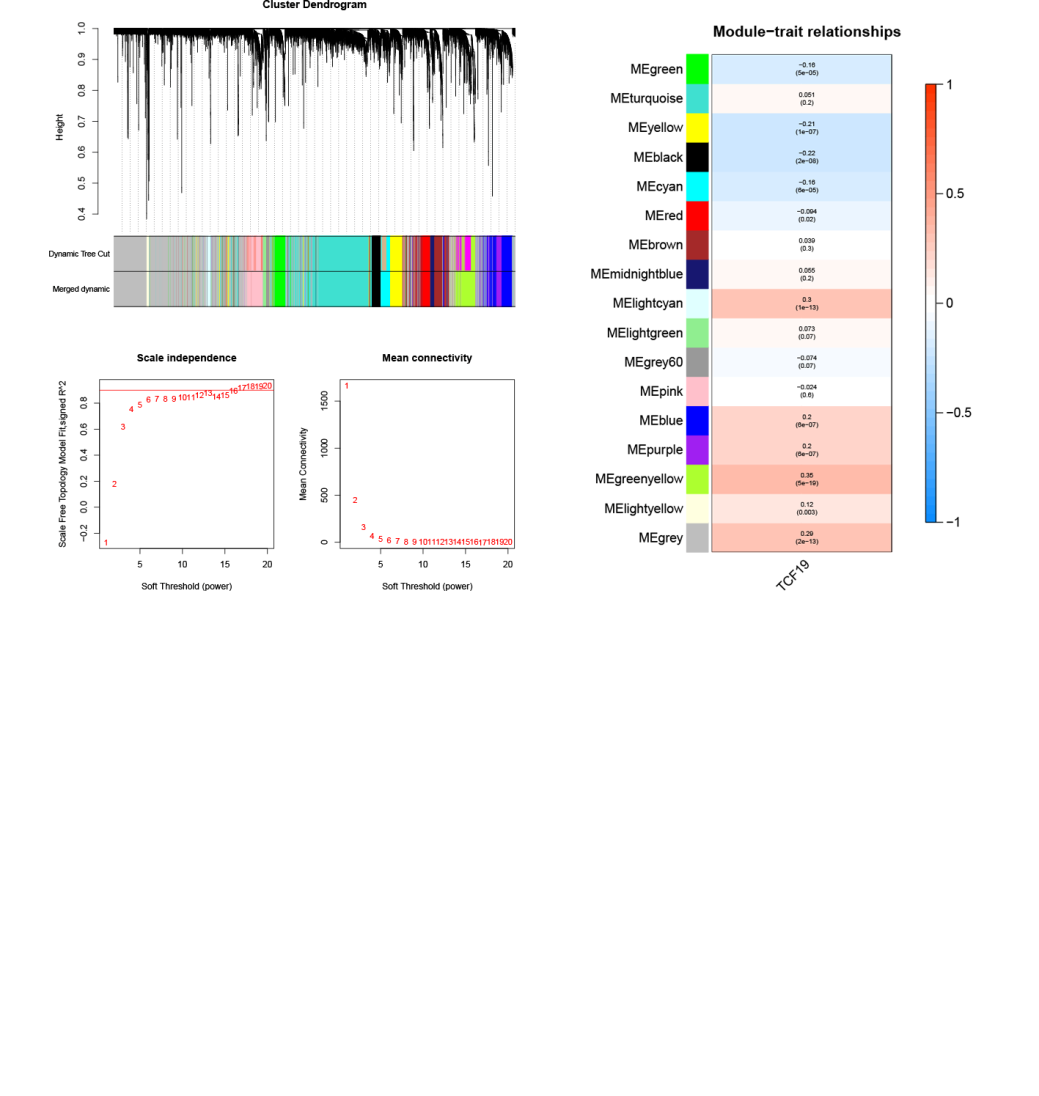


**SUPPLEMENTARY FIGURE 3** The WGCNA analysis of TCF19 in pan-cancer.


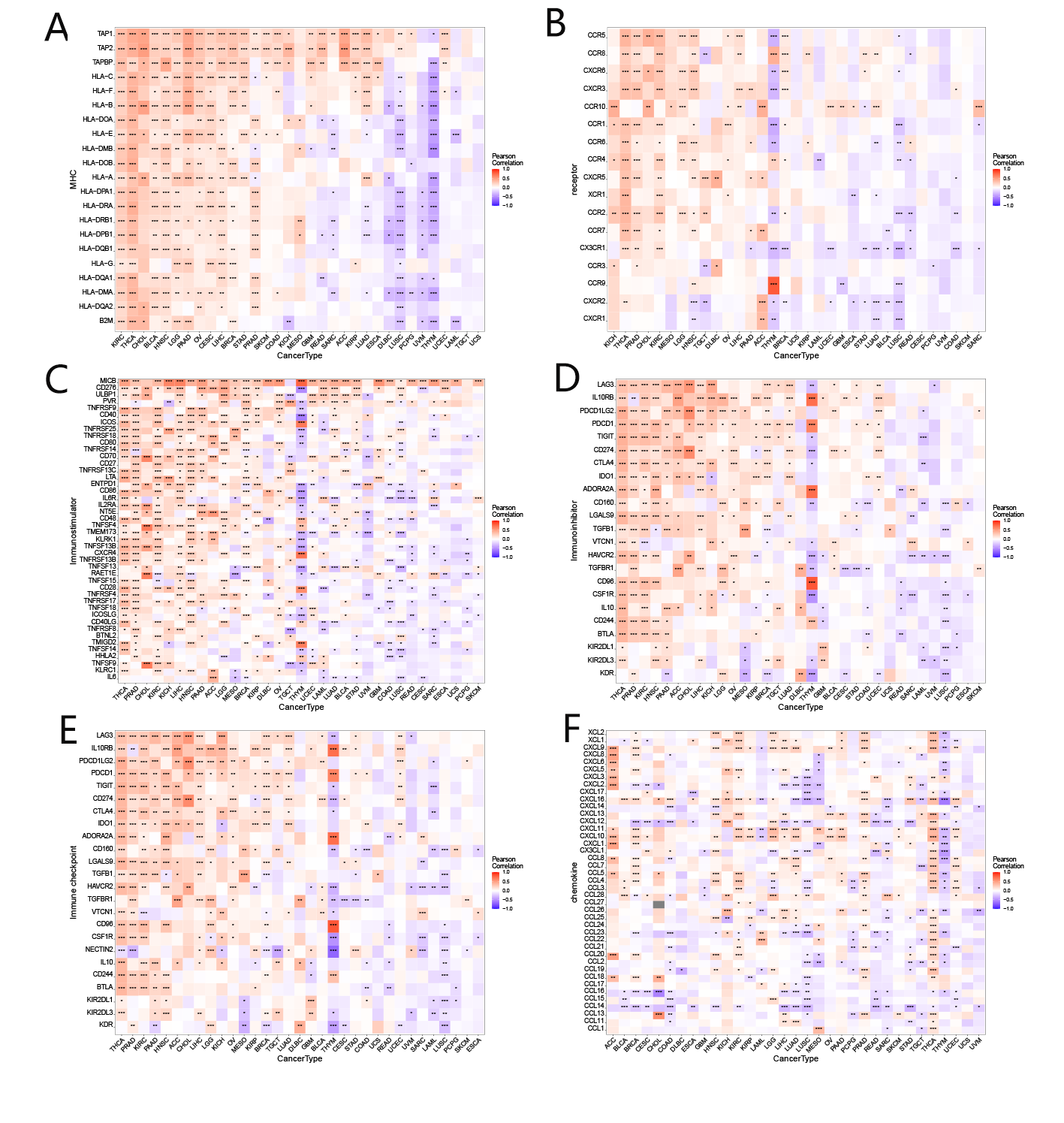


**SUPPLEMENTARY FIGURE 4(A-F)** The relationship between TCF19 expression and 33 tumor immune-related genes (Genes analyzed include MHC, immune activators, immune suppressors, chemokines and chemokine receptor proteins).


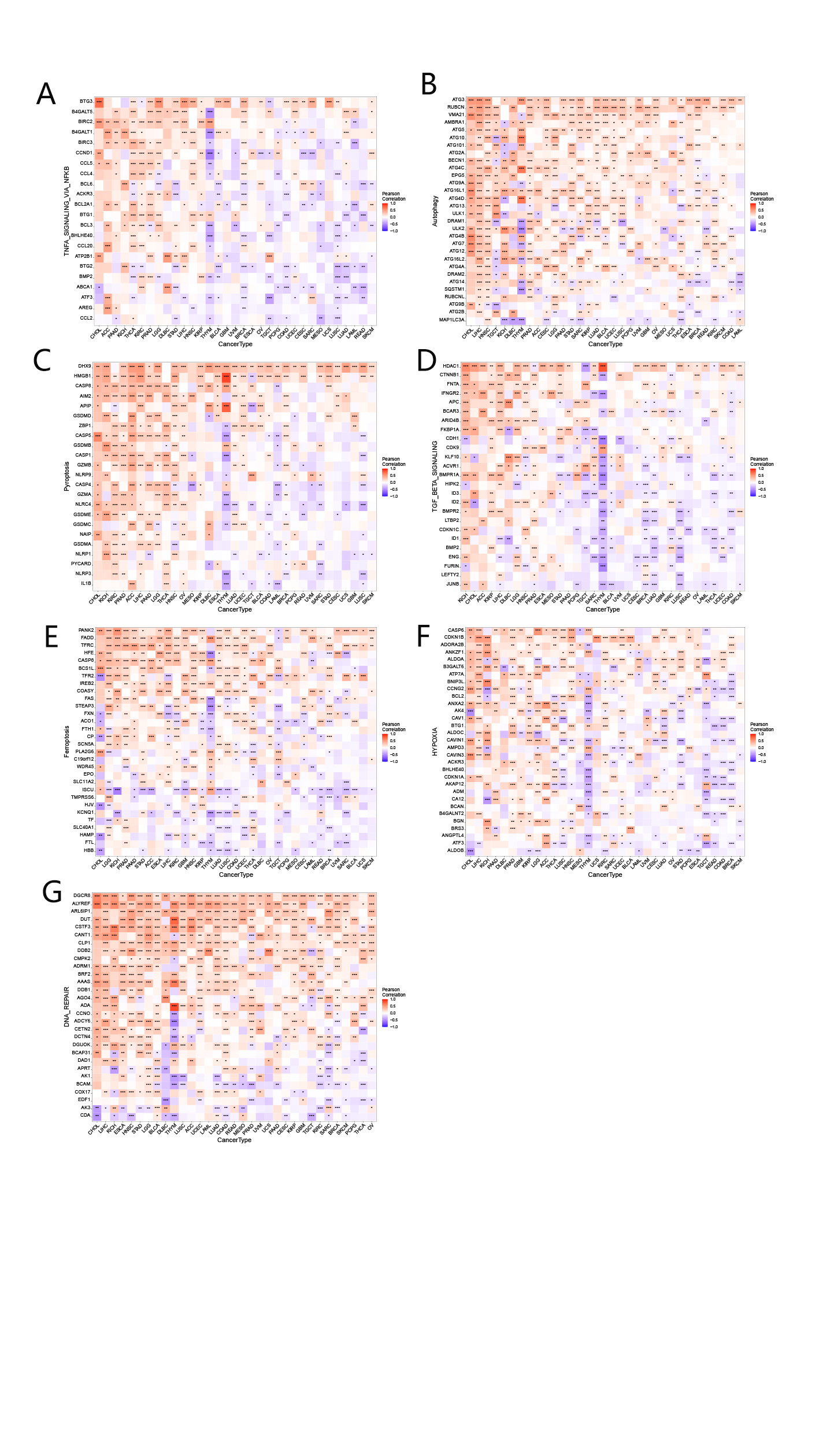


**SUPPLEMENTARY FIGURE 5(A-G)** The association between TCF19 and common tumor-associated regulatory genes (Such as : TGF BETA SIGNALING, TNFA SIGNALING, Hypoxia, Scorch Death, DNA Repair, Autophagy genes, Iron Death-Related genes).





**SUPPLEMENTARY FIGURE 6** The analysis of the relationship between TCF19 and the sensitivity of common antitumor drugs.
